# A rare cause of proximal intestinal obstruction in adults - annular pancreas: a case report

**Published:** 2011-12-18

**Authors:** Bouassida Mahdi, Sassi Selim, Touinsi Hassen, Mighri Mohamed Mongi, Chtourou Mohamed Fadhel, Chebbi Fathi, Sassi Sadok

**Affiliations:** 1 Department of surgery, Mohamed Tahar Maamouri hospital, Nabeul, Tunisia

**Keywords:** Annular pancreas, duodenal stenosis, bypass

## Abstract

Annular pancreas is a rare congenital anomaly characterized by the presence of ectopic pancreatic tissue surrounding the descending part of the duodenum. It is one of the few congenital anomalies of the gastrointestinal tract which can produce symptoms late in life. In adults, the factors initiating symptoms are recurrent pancreatitis, duodenal stenosis at the site of the annulus, or duodenal or gastric ulceration. We report a new case involving a 24-year-old woman hospitalised for epigastric pain, nausea and vomiting. Radiological examination was consisted with an annular pancreas. At operation a complete obstruction of the second part of the duodenum was found, caused by an annular pancreas, no other congenital anomaly of the intra-abdominal organs was noted. A gastroenterostomy was performed.

## Introduction

Annular pancreas is a rare congenital anomaly due to an abnormal fusion between the tip of the ventral pancreatic bud and the duodenum. In actuality, annular pancreas is diagnosed with nearly equal frequency in children and adults. It is one of the few congenital anomalies of the gastrointestinal tract which can produce symptoms late in life. In adults, the factors initiating symptoms are recurrent pancreatitis, duodenal stenosis at the site of the annulus, or duodenal or gastric ulceration.

One new case of annular pancreas is presented, which is treated surgically. The etiology, symptoms and particularly the diagnosis in adults with annular pancreas are analyzed.

## Case report

**Figure 1:**
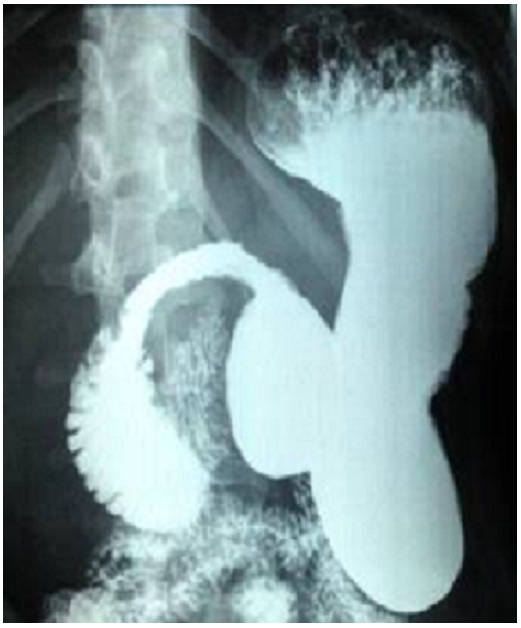
Contrast barium radiography showing an obstructed third part of the duodenum. Proximal to the obstruction a dilated duodenal bulb and stomach was found: classically known as “double bubble” sign

A 24-year old man presented complaining of repeated episodes of mild epigastric pain, nausea and vomiting. The symptoms had been present for more than 2 years, but had become more frequent in the last few months. A radiograph of the stomach-duodenum revealed stenosis of the second part of the duodenum (Figure 1). The scan showed an annular pancreas encircling thee duodenum. Exploratory laparotomy revealed the presence of ectopic pancreatic tissue encircling the whole of the duodenum between its first and second parts. Exploration of other intra-abdominal organs revealed no other congenital anomalies. A gastroenterostomy was performed. The patient remains in good general health 6 months after the operation.

## Discussion

403 reports of annular pancreas have been published [1]. It is one of the few congenital abnormalities of the gastrointestinal tract which can produce symptoms late in life [2]. This phenomenon has not been fully explained until now.

Three developmental theories are favored for formation of an annular pancreas: hypertrophy of both the ventral and dorsal analage, adhesion of a portion of the ventral analage to the duodenum before migration, or fusion of aberrant pancreatic tissue from the duodenum. The resulting malformation usually consists of a fiat band of pancreatic tissue encircling the second portion of the duodenum.

The ring of normal pancreatic tissue produces symptoms when it obstructs the duodenum. It has been estimated that only about 33% of the cases are symptomatic. 50% of patients present in the pediatric age group, 86% of these present in the neonatal period. In adults, annular pancreas usually presents between age 20 and 50 and is most commonly associated with abdominal pain and gastric outlet obstruction, secondary to duodenal stenosis. Additional presentations including pancreatitis, peptic ulcer disease and obstructive jaundice have been reported. Annular pancreas associated with a pancreatic tumor has also been reported. The diagnosis is usually made with computed tomography scanning and confirmed with upper gastrointestinal contrast fluoroscopy [3].

The treatment of annular pancreas is surgical, with various procedures being used: gastro-enteral anastomosis and truncal vagotomy; latero-lateral anastomosis of the first part of the duodenum with the jejunum; latero-lateral anastomosis of the pyloroduodenal portion with the jejunum; duodenoduodenal anastomosis; duodeno-jejunal anastomosis with Roux en Y loop; and separation and resection of the annular pancreas. Gastroenterostomy with truncal vagotomy is performed where an ulcer is also present, while separation of the annulus is associated in 50% of the cases with serious complications. Resection of an annular pancreas is contraindicated when there is danger of postoperative pancreatitis or fistula, the possibility of a co-existing mucosal diaphragm in the duodenum or erosion of the duodenal wall by the pancreatic ring [4]. The results of previously reported operations are satisfactory, especially the gastrenterostomy and the duodenoduodenal anastomoses, which are simple operations performed frequently and have the best results.

## Conclusion

Annular pancreas is a rare malformation that can present in infancy or adulthood. Most affected patients present with signs and symptoms of a proximal intestinal obstruction. After evaluation, these patients can be managed safely with surgical bypass of the annulus to restore intestinal continuity.
